# Intracellular Antioxidant Activity of Biocompatible Citrate-Capped Palladium Nanozymes

**DOI:** 10.3390/nano10010099

**Published:** 2020-01-03

**Authors:** Mauro Moglianetti, Deborah Pedone, Gayatri Udayan, Saverio Francesco Retta, Doriana Debellis, Roberto Marotta, Antonio Turco, Simona Rella, Cosimino Malitesta, Giulia Bonacucina, Elisa De Luca, Pier Paolo Pompa

**Affiliations:** 1 Nanobiointeractions & Nanodiagnostics, Center for Biomolecular Nanotechnologies, Istituto Italiano di Tecnologia, via Barsanti, 73010 Arnesano, Lecce, Italy; deborah.pedone@iit.it (D.P.); gayatriudayan@gmail.com (G.U.); 2 Department of Engineering for Innovation, University of Salento, Via per Monteroni, 73100 Lecce, Italy; 3 Department of Clinical and Biological Sciences, University of Torino, 10043 Orbassano (Torino), Italy; francesco.retta@unito.it; 4 Electron Microscopy Laboratory, Nanochemistry Department, Istituto Italiano di Tecnologia, via Morego 30, 16163 Genova, Italy; doriana.debellis@iit.it (D.D.); roberto.marotta@iit.it (R.M.); 5 Dipartimento di Scienze e Tecnologie Biologiche e Ambientali (Di.S.Te.B.A.), Università del Salento, via Monteroni, 73100 Lecce, Italy; antonio.turco@unisalento.it (A.T.); simona.rella@unisalento.it (S.R.); cosimino.malitesta@unisalento.it (C.M.); 6 School of Pharmacy, Via Gentile III da Varano, University of Camerino, 62032 Camerino, Italy; giulia.bonacucina@unicam.it; 7 Nanobiointeractions & Nanodiagnostics, Istituto Italiano di Tecnologia, via Morego 30, 16163 Genova, Italy

**Keywords:** palladium nanoparticles, aqueous synthesis, nanozymes, oxidative stress, antioxidants, scavengers, toxicology, SEI-XPS

## Abstract

A method for the aqueous synthesis of stable and biocompatible citrate-coated palladium nanoparticles (PdNPs) in the size range comparable to natural enzymes (4–8 nm) has been developed. The toxicological profile of PdNPs was assessed by different assays on several cell lines demonstrating their safety in vitro also at high particle concentrations. To elucidate their cellular fate upon uptake, the localization of PdNPs was analyzed by Transmission Electron Microscopy (TEM). Moreover, crucial information about their intracellular stability and oxidation state was obtained by Sputtering-Enabled Intracellular X-ray Photoelectron Spectroscopy (SEI-XPS). TEM/XPS results showed significant stability of PdNPs in the cellular environment, an important feature for their biocompatibility and potential for biomedical applications. On the catalytic side, these PdNPs exhibited strong and broad antioxidant activities, being able to mimic the three main antioxidant cellular enzymes, i.e., peroxidase, catalase, and superoxide dismutase. Remarkably, using an experimental model of a human oxidative stress-related disease, we demonstrated the effectiveness of PdNPs as antioxidant nanozymes within the cellular environment, showing that they are able to completely re-establish the physiological Reactive Oxygen Species (ROS) levels in highly compromised intracellular redox conditions.

## 1. Introduction

Palladium nanoparticles (PdNPs) represent a promising player in nanomedicine and nanodiagnostics, due to their efficient and broad catalytic activities [1,2,3,4,5,6,7] that are opening the way to a variety of applications [8]. In particular, their use as catalyst/nanozyme for chemical reactions of therapeutic relevance within cellular environment would be of great interest. Up to now, however, only few promising reports have proposed the use of PdNPs, showing that they can catalyze the conversion of inactive prodrugs into toxic drugs directly within cells, thus enabling local chemotherapy [1,9,10,11,12,13]. The catalytic properties of PdNPs confer them the ability to act as artificial antioxidant nano-enzymes (nanozymes) [2,3,4,14,15] for potential applications in the study of the pathogenesis and treatment of oxidative stress-related diseases. Preliminary data suggest that PdNPs possess radical scavenging activity similar to that of natural enzymes, namely catalase (CAT) [14,16], peroxidase (HRP) [3,16,17], and superoxide dismutase (SOD) [14,15], but these studies are limited to a few cases, in a cell-free environment, and do not provide a clear picture of their structure-function relationship [18]. Moreover, despite such interesting features, the biomedical use of PdNPs is still limited, partially due to conflicting data about their toxicity [19,20,21,22,23,24,25,26,27]. In this regard, the presence of synthesis by-products, endotoxins, contaminations, and surface functionalities has a strong influence and could be the reason of the observed toxicity in many reports [28]. Therefore, the determination of the toxicological profile of the nanomaterial is still missing, even though it is crucial for the applications of Pd in the field of nanomedicine [29]. A clearer picture of the toxicological profile of PdNPs would promote the exploitation of their catalytic activities in the medical field for developing novel nanocatalysts or drug delivery nanosystems with therapeutic properties.

We developed a new protocol of synthesis preferentially based on biocompatible, green, or biogenic reagents and aqueous environment to obtain monodisperse and biocompatible PdNPs, only covered by citrate molecules. Two different sizes, 4 and 8 nm, were synthesized to match the size range of natural enzymes. With this highly purified material, we performed a systematic toxicity assessment, demonstrating their safety in vitro up to high particle concentrations. Inductively Coupled Plasma Atomic Emission spectroscopy (ICP-AES) and Transmission Electron Microscopy (TEM) along with a recently developed technique, namely Sputtering-Enabled Intracellular X-Ray Photoelectron Spectroscopy (SEI-XPS) [30], were used to investigate the PdNP cellular uptake and fate. After a detailed characterization on the antioxidant properties of PdNPs, we proved their potential to function as antioxidant nanozymes in a cell line model of a human oxidative stress-related disorder.

## 2. Results 

### 2.1. Aqueous Synthesis of PdNPs and Physico-Chemical Characterization

The application of nanomaterials in nanomedicine and nanodiagnostics requires a strict control of their physico-chemical properties, achievable by a careful design of the synthetic procedure [5,7,28,31,32,33,34,35,36,37,38,39,40]. To this purpose, we developed a new protocol for the synthesis of PdNPs that provides low polydispersity along with size tunability, combined with reagents known to be biocompatible, green, or easy to remove after synthesis. All syntheses were performed using BioXtra and BioUltra pure reagents in aqueous environment, with extensive post-synthesis purification. This is important for the application of these NPs, as it should guarantee the absence of toxicity factors other than the material per se, i.e., contaminants, endotoxins, and reaction by-products [34,39,41,42].

The main reagent in the synthesis was sodium citrate, which plays a role in both reducing and stabilizing the nanoparticles. As citrate molecules have carboxylic and hydroxyl groups, they weakly bind the surface of PdNPs stabilizing the particles in solution against aggregation. Moreover, they only partially cover NP surface and can easily be removed. In this way, a “naked” surface, a pivotal aspect for catalysis/nanozyme applications, can be obtained without major cleaning processes, unlike synthetic protocols based on polymers or strong surfactants [41]. Furthermore, the proposed procedure tries to strictly follow the “green” synthesis requirements, as it uses water as solvent and only cytocompatible compounds, an important feature considering the strong need to develop low environmental-impact synthetic protocols of nanomaterials [41].

**Figure 1:**
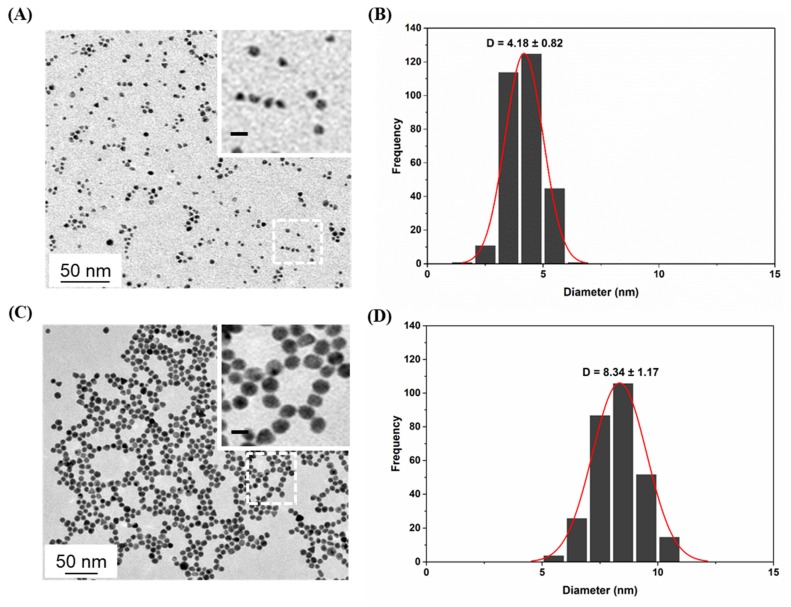
Representative Transmission Electron Microscopy (TEM) micrographs and nanoparticle (NP) size distribution analyses of Pd4 (**A**,**B**) and Pd8 (**C**,**D**). Scale bars in the insets: 10 nm.

Monodisperse citrate-capped PdNPs with a diameter of 4 nm (Pd4) were obtained by combining sodium citrate and sodium borohydride, in order to promote a quick and uniform growth of the nanomaterial with spherical geometry (Figure 1A,B). 8 nm PdNPs (Pd8) (Figure 1C,D) were obtained by seed-growth method using only sodium citrate, formic acid, and L-ascorbic acid with a reaction time of circa 10 min. The size tunability was achieved also by the control of the reaction rate [43]: By dissolving the PdCl_2_ precursor in different acidic media (0.1 M HNO_3_ or CH_3_COOH), different Pd complexes are formed with different stability and, hence, reduction kinetics [34]. The synthesis was also scaled-up by employing a microwave reactor, with multi-vessel setup, achieving a production yield of circa 50/60 mg PdNPs per hour.

PdNPs were characterized by TEM imaging, which revealed the formation of spherical PdNPs with low polydispersity and size distributions centered around 4 and 8 nm (Figure 1). The inset of Figure 1 shows that the nanoparticles were well separated also after the deposition on the TEM grids, despite being only covered by citrate molecules. To the best of our knowledge, this is the first synthetic protocol that achieves the production of monodisperse PdNPs with size tunability, using only sodium citrate as a capping agent in aqueous environment [5,44,45,46]. This is particularly important, as the majority of PdNP syntheses described in the literature requires the use of organic solvents and difficult-to-remove capping agents (polymers, surfactants, and/or thiolated molecules), two features that can strongly affect the catalytic properties and the toxicological profile of the nanomaterial.

Before biological assessments, the Limulus test [47] was used to confirm the absence of endotoxins in the solution.

### 2.2. Toxicity Assessment of PdNPs

A careful study of the toxicological profile and intracellular fate of NPs is crucial to uncover their biomedical potential [28,31,33]. Although there are several studies reported in the literature [1,9,10,13,14,19,20,21,22,23,24,25,26,27,48,49,50,51,52,53,54], there is a lack of conclusive data about the biocompatibility of PdNPs. The results are rather conflicting, likely due to the high variety of PdNP synthetic protocols, dimensions, shapes, surface capping agents, purity, cellular lines used for toxicity tests, and the variability of experimental conditions. In vitro and in vivo studies reported cytocompatibility for pristine PdNPs and PdNPs coated with biocompatible compounds [14,51,54,55], while others reported cytotoxic effects, as a consequence of Pd ion release [25] or Reactive Oxygen Species (ROS) overproduction [27]. Here, we performed a systematic in vitro assessment on monodisperse and pure citrate-capped Pd4 and Pd8, by monitoring their effects on cellular metabolism, membrane integrity, and ROS levels (through Water Soluble Tetrazolium salt (WST-8), Lactate Dehydrogenase (LDH), and 2′,7′-Dichlorofluorescein diacetate (DCFH-DA) assays), and evaluating their cellular uptake by Inductively Coupled Plasma Atomic Emission spectroscopy (ICP-AES) and Transmission Electron Microscopy (TEM). Moreover, to characterize NP stability/degradation and oxidation state within the cellular environment, we exploited a recently developed technique, namely Sputtering-Enabled Intracellular X-Ray Photoelectron Spectroscopy (SEI-XPS) [30].

**Figure 2:**
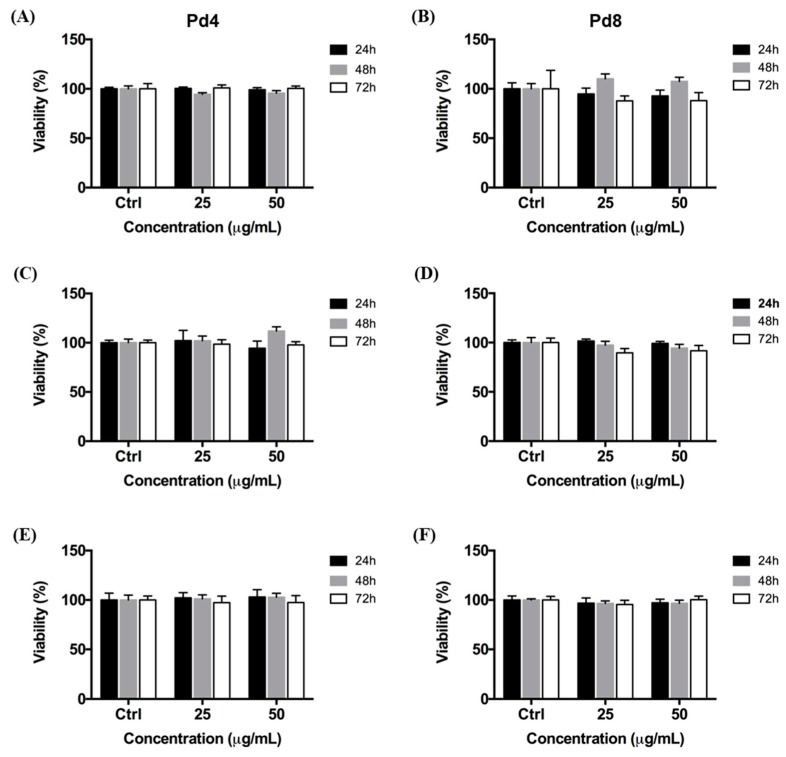
Viability of HeLa (**A**,**B**), MCF-7 (**C**,**D**), and Caco-2 (**E**,**F**) cells after treatment with increasing concentrations of Pd4 (**A**,**C**,**E**) and Pd8 (**B**,**D**,**F**) for 24, 48, and 72 h. Treated cells viability is expressed as a percentage relative to untreated cells (Ctrl). Results are reported as mean ± SD. The experiments were repeated three times and performed in triplicate.

We evaluated the impact of increasing concentrations of Pd4 and Pd8 by performing WST-8 cytotoxicity assays after 24, 48, and 72 h of treatment (Figure 2). We observed that, independently of their size, PdNPs do not reduce viability of HeLa (Figure 2A,B), MCF-7 (Figure 2C,D), and Caco-2 (Figure 2E,F) cells, even at high NP concentration (50 μg/mL). These data are in line with other literature reports showing that PdNPs coated by biocompatible materials do not cause cellular damage in vitro [14,56].

**Figure 3:**
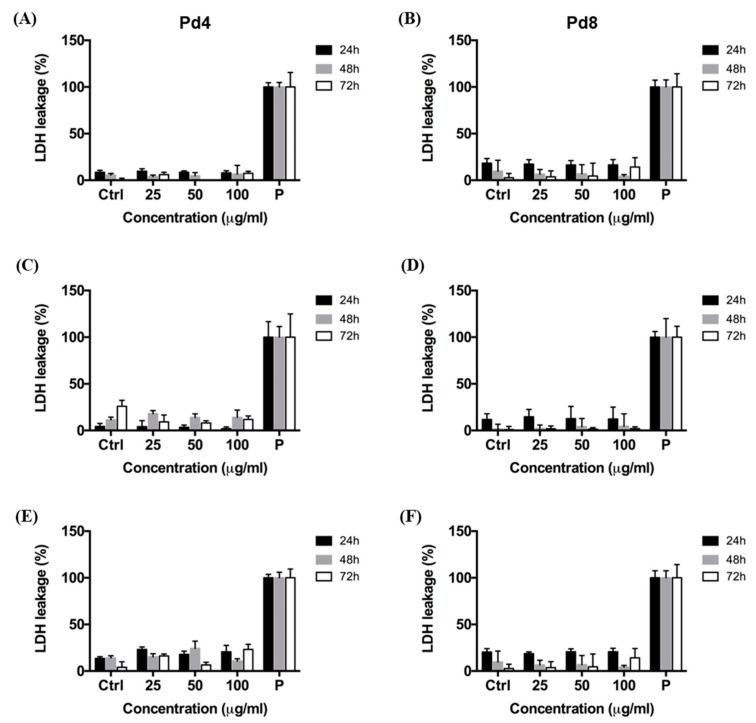
Lactate Dehydrogenase (LDH) release in HeLa (**A**,**B**), MCF-7 (**C**,**D**), and Caco-2 (**E**,**F**) cells after treatment to increasing doses of Pd4 (**A**,**C**,**E**) and Pd8 (**B**,**D**,**F**) for 24, 48, and 72 h. LDH leakage of NP treated cells is expressed as a percentage relative to cells exposed to lysis buffer (P). Results are reported as mean ± SD. The experiments were repeated three times and performed in triplicate.

To strengthen the information regarding the toxicological profile of PdNPs, we assessed the integrity of cellular membranes by lactate dehydrogenase (LDH) assay, since it has been previously reported that cellular treatment with metal nanoparticles may induce membrane damage [57,58,59], even without inducing detectable alteration of mitochondrial activity. We found that the membrane integrity of HeLa, MCF-7, and Caco-2 cells was not affected by exposure to Pd4 and Pd8, also by using higher particle concentration (up to 100 μg/mL) (Figure 3).

**Figure 4:**
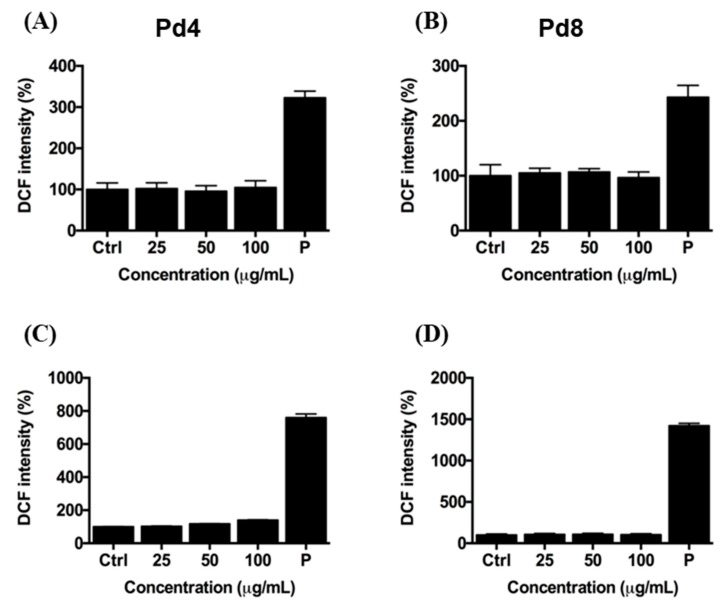
Reactive Oxygen Species (ROS) levels in HeLa (**A**,**B**) and Caco-2 (**C**,**D**) cells, after treatment with increasing concentrations of Pd4 (**A**,**C**) and Pd8 (**B**,**D**) for 24 h, detected by DCFH-DA assay. ROS amount of NP-treated cells is reported as a percentage relative to cells exposed to 1 M H_2_O_2_ for 10 min (P). Results are reported as mean ± SD. The experiments were repeated three times and performed in triplicate.

In addition, to evaluate possible alteration of ROS homeostasis due to PdNP internalization, we quantified the intracellular levels of ROS by 2′,7′-Dichlorofluorescein diacetate (DCFH-DA) assay [60,61], as in some studies oxidative stress has been described as one of the processes of PdNP cellular toxicity [24,27]. HeLa and Caco-2 cells were treated for 24 h with high doses of PdNPs (up to 100 μg/mL) (Figure 4). We observed that, regardless of their size and concentration, PdNPs do not alter ROS intracellular basal levels, thus, providing a further hint of their biocompatibility. Taken together, these results show that our citrate-capped PdNPs with size below 10 nm do not affect cellular viability, membrane integrity of cells, and ROS homeostasis, also at high concentrations.

**Figure 5:**
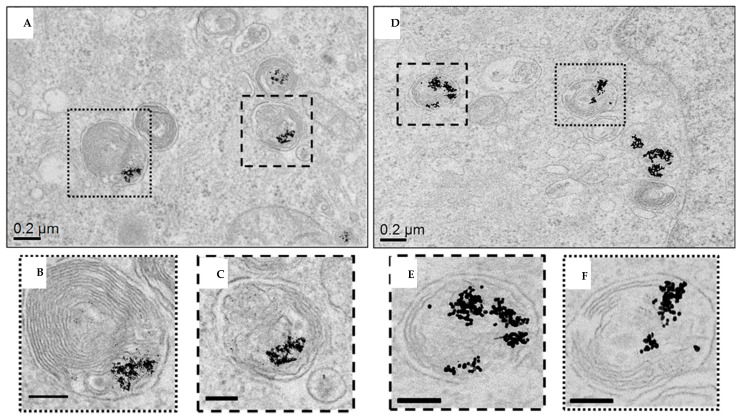
TEM analysis of HeLa cells exposed to Pd4 (**A**–**C**) and Pd8 (**D**–**F**) for 24 h. Low magnification projection images of late endosome/phagosome compartments containing Pd4 and Pd8 are reported in (**A**,**D**). (**B**,**C**) and (**E**,**F**) are higher magnification of the boxed region in (**A**,**D**). Scale bars in (**B**,**C**) and in (**E**,**F**) are 100 nm.

The study of NP internalization and their intracellular fate is of fundamental relevance to understand the mechanisms of their toxicity. To analyze the uptake of PdNPs, we measured by ICP-AES the content of Pd in HeLa and Caco-2 cells exposed to 50 μg/mL of Pd4 for 24 h (Supplementary Table S1). The results show an abundant uptake of PdNPs, in line with previous reports for other metal NPs [39,62]. In particular, we found a cell type-dependent internalization of nanoparticles, because Caco-2 cells endocytosed circa one tenth of the particles internalized by HeLa cells (Supplementary Table S1), likely due to their different cell body dimensions. This is in qualitative agreement with previous studies with other metal NPs [39,63]. We then imaged by TEM the subcellular localization of PdNPs in HeLa cells, upon exposure to Pd4 and Pd8 for 24 h. As shown in Figure 5 and in Supporting Figure S1, NPs were internalized in cells by the endo-lysosomal pathway and confined into late endosomal and/or lysosomal compartments. TEM micrographs mostly show small particle aggregates dispersed into endo/lysosomes, with some single NPs also present (especially for Pd4). Interestingly, analyzing the size of the internalized particles, we observed that PdNPs typically maintain their pristine size after 24 h. This suggests that the particles do not undergo significant dissolution when exposed to the acidic and degradative environment of lysosomes, unlike for instance silver NPs [40,57]. In light of these results, the biocompatibility of PdNPs could be ascribed to the stability of the material (i.e., resistance to the acidic corrosion) in the cellular environment and absence of significant Pd ion release. Moreover, considering the interplay of particle physico-chemical properties with surface coatings and possible contaminants in determining NP toxicological profile, our results confirm the purity of the nanomaterial synthesized. This is a fundamental aspect for the introduction of new nanozymes in nanobiomedicine.

### 2.3. PdNP Cellular Fate by SEI-XPS

**Figure 6:**
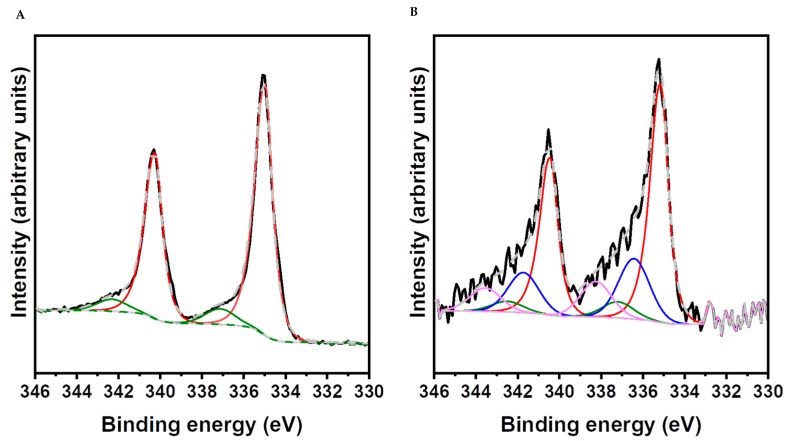
(**A**) High-resolution X-Ray Photoelectron Spectroscopy (XPS) spectra of the Pd 3D region with relative fits of PdNPs after deposition on a glass substrate. Data are shown in black and their fits are as follows: Pd(0) in red, Pdox_pr_ in green, and fitted peaks sums in gray dashed lines. (**B**) High-resolution XPS spectra of the Pd 3D region for HeLa cells after treatment with PdNPs for 48 h after 300 s of argon sputtering time. Data are shown in black and their fits are as follows: Pd(0) in red, Pdox_1_ in blue, Pdox_2_ in pink, Pdox_pr_ in green, and fitted peaks sums in gray dashed lines.

Among the recently developed tools to assess the fate of nanomaterials in biological systems, SEI-XPS represents a valuable technique, as it permits complete chemical assessment of inorganic nanoparticle fate within cells, i.e., their in-situ stability/degradation and oxidation state, with nanoscale vertical resolution [30]. Figure 6A reports a representative high-resolution spectrum of the Pd 3D region of PdNPs before incubation in cell culture medium. The signal is split in two peaks with well-defined ratio, due to the spin–orbit coupling effects. These spectra can be fitted in two pairs of doublets. The signal at 335.0 eV can be ascribed to zero valent palladium, while the small peak at 337.1 eV is due to Pd oxidized species in the pristine material (Pd-ox_pr_), in agreement with previous studies [64]. Such low percentage of oxidized species of Pd is likely due to some ageing of the material once exposed to air for several days before the measurement. XPS characterization was directly performed on HeLa cells, previously incubated with PdNPs for 48 h. Supporting Figure S2a,b shows a representative survey spectrum, in which the signals of the chemical elements constituting the cell membrane, such as C, O, N, P, Na, and Cl, are clearly visible. Si signals are weak, demonstrating that the glass support was almost completely coated by cells on the measurement area (circa 700 × 300 μm^2^). Moreover, Pd 3d signals were not present (Supporting Figure S2c), suggesting that there are not PdNPs on the exterior surface of the cellular layer (~10 nm), corresponding to the cellular membrane or the cytosol close to it. This result is due to the efficacy of the extensive washing protocol applied before the measurement that permits the complete removing of NPs weakly bound to the cell surface. To further investigate intracellular NP distribution, argon sputtering was used to etch the surface layer and expose the intracellular environment. In Supporting Figure S2d,e a survey spectrum collected following argon sputtering (300 s) is reported. The intensities of photoelectron lines related to Si, Cl, P, and Na became relevant, as the intracellular environment is exposed due to the etching performed by argon sputtering. Furthermore, two peaks at binding energies (BE) of 335.2 and 340.6 became evident (Supporting Figure S2f) and were ascribed to Pd 3D photoemission. High-resolution XPS peaks of the Pd 3d obtained from cells exposed to PdNPs were fitted, as shown in Figure 6B. Pd 3D peaks exhibited a broadening with respect to pristine NPs (Figure 6A) and at least two more spin−orbit pairs of components became evident. These spectra related to Pd within cells could be fitted into four pairs of doublets with binding energies for Pd 3d_5/2_ of 335.0 ± 0.2, 337.1 ± 0.2, 336.3 ± 0.2, and 338.2 ± 0.2 [65]. The relative intensities of the four components were quantified to be 60.1 ± 0.2%, 6.3 ± 0.2%, 20.2 ± 1.3, and 13.4 ± 1.3%, respectively. The two new signals at 336.3 and 338.2 eV were ascribed to Pd oxidized species (Pd-ox_1_ and Pd-ox_2_, likely Pd(II) and Pd(IV)) due to the NP surface oxidation, which was elicited by the intracellular environment. Indeed, we verified that argon sputtering does not modify the nanomaterial, as longer sputtering times did not alter the proportion between the four components. This confirms that the cell etching treatment does not induce detectable modifications of the oxidation state of PdNPs (Supporting Figure S3). The overall percentage of the oxidized Pdox_1_ and Pdox_2_ species, due to the endo-lysosomal environment, was approximately ~33% and was most likely due to the oxidation of the superficial Pd atoms after the binding of thiolated molecules (e.g., glutathione), highly present within the cellular environment. However, PdNPs did not undergo dissolution, as also proven by the TEM analysis showing that NPs maintain their pristine size. The percentage of oxidized material was comparable to the one previously observed for PtNPs, while AgNPs were found to be completely oxidized and dissolved after 48 h within the cellular environment [30].

These results underline that PdNPs can withstand the harsh endo-lysosomal environment without showing significant dissolution. This is a crucial information for the evaluation of the cytotoxic effect of NPs, as the release of toxic ions within the cells represents one of the main mechanisms behind the toxicity of the nanomaterials [57,66].

### 2.4. ROS Scavenging Activities of PdNPs

Even though it is known that PdNPs possess remarkable catalytic activity [1,2,3,4,14,15,16,17], a detailed analysis of such properties has not been performed yet [14,18,20]. Furthermore, to harness the full potential of their antioxidant activity, an accurate control of the physico-chemical properties of the nanomaterial is necessary, in order to engineer enzyme-mimetics for the scavenging of ROS in the body [2,67,68].

**Figure 7:**
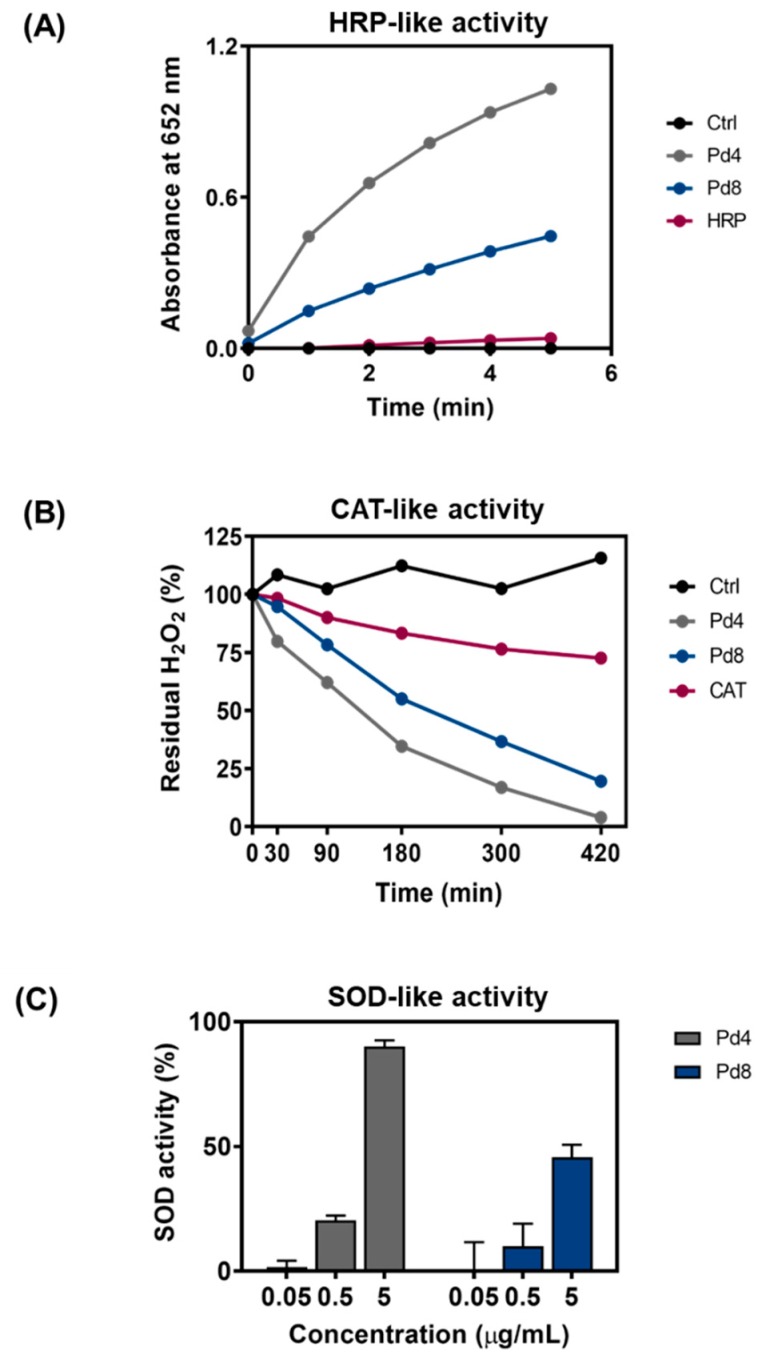
PdNP antioxidant activity. (**A**) Time-dependent absorbance signals at 652 nm of 3,5,3′,5′-tetramethylbenzidine (TMB) (0.5 mM) after incubation at room temperature with 0.05 μg/mL of Pd4 (grey), Pd8 (blue), natural enzyme (purple), and water as control (black) in the presence of 200 mM H_2_O_2_ in pH 4.7 acetate buffer. (**B**) Time-dependent degradation of 40 μM H_2_O_2_ at room temperature after incubation with 0.5 μg/mL of Pd4 (grey), Pd8 (blue), natural enzyme (purple), and water as control (black). (**C**) Dose-dependent superoxide dismutase (SOD) mimetic activity of PdNPs. SOD activity of Pd4 (grey) and Pd8 (blue) at increased concentrations.

A major role to control ROS homeostasis in biological systems is played by three antioxidant enzymes, i.e., peroxidase (HRP), catalase (CAT), and superoxide dismutase (SOD). Here, we first evaluated the ability of PdNPs to mimic the antioxidant defense system in a cell-free environment and the effect of the particle size on their performance as antioxidants. HRP is the biological enzyme responsible for the decomposition of hydrogen peroxide (H_2_O_2_) into water, through the oxidation of antioxidant molecules, such as glutathione. We evaluated the ability of PdNPs to act as HRP mimics by monitoring the oxidation rate of 3,5,3′,5′-tetramethylbenzidine (TMB) in presence of H_2_O_2_. We found that both Pd4 and Pd8 are able to efficiently catalyze the oxidation of TMB, even at very low concentrations (Figure 7A). Interestingly, a size-dependent catalytic activity was observed, in qualitative agreement with a previous study on PtNPs [39]. Pd4 were found to be significantly more efficient than Pd8 in catalyzing the TMB oxidation, due to their higher surface-to-volume ratio. Remarkably, both NPs oxidized TMB with comparable or higher performance than the natural HRP enzyme. This is likely due to the ability of Pd nanozymes to operate in a wider range of conditions, such as non-physiological temperature, high H_2_O_2_ concentrations, and acidic pH (required for the TMB assay), which partly inhibit the activity of the biological enzyme [39]. Their lower sensitivity to the external conditions (broader range of pH and temperature) suggests the superior versatility of Pd nanozymes over the natural HRP, as artificial peroxidases in biological applications.

The CAT enzyme catalyzes the reduction of H_2_O_2_ into water and molecular oxygen. We evaluated CAT mimetic activity of PdNPs by measuring residual H_2_O_2_ after different incubation times (Figure 7B). We observed a time-dependent reduction of H_2_O_2_ in presence of PdNPs or natural CAT enzyme. Pd4 nanozymes catalyze the reaction at a higher rate than Pd8, in line with their HRP activity. Both Pd4 and Pd8 showed higher performance than the natural CAT enzyme. Finally, we investigated the SOD-like antioxidant activity of PdNPs. We assayed the ability of Pd4 and Pd8 at different concentrations to catalyze the dismutation of O^2−^ into molecular oxygen and H_2_O_2_ (Figure 7C). Results showed a dose- and size-dependent SOD-mimicking activity of NPs. Both Pd4 and Pd8 proved to be more efficient than the SOD enzyme. In particular, Pd4 reached the same enzymatic activity of 100 U mL^−1^ (12 μM) SOD [69] at considerably lower concentration (0.018 μM).

As further demonstration of the superior antioxidant properties of PdNPs, their ability to block the production of the radical cation 2,2′-azinobis(3-ethylbenzothiazoline-6-sulfonic acid) radical cation (ABTS^+^) was compared to that of 6-hydroxy-2,5,7,8-tetramethylchroman-2-carboxylic acid (Trolox). Our results show that the scavenging ability of PdNPs is higher than that of the antioxidant compound, with longer lasting performance (Supporting Figure S4).

The catalytic performance of Pd4 and Pd8 seems to be comparable to those of PtNPs with similar size, shape, and coating [39], which are well recognized in nanomedicine for their high catalytic activity. This is an interesting point, as it extends the list of nanomaterials that can be potentially exploited for catalysis within cells, or for developing innovative in vitro diagnostic tools. These results also provide evidence that our biocompatible PdNPs possess very efficient and broad antioxidant activities and, hence, they represent a good platform to engineer highly efficient artificial antioxidant enzymes as new therapeutic tools for the treatment of pathologies related to redox imbalance.

### 2.5. ROS Scavenging Effects of PdNPs in a Cellular Model of Oxidative Stress-Related Disease

**Figure 8:**
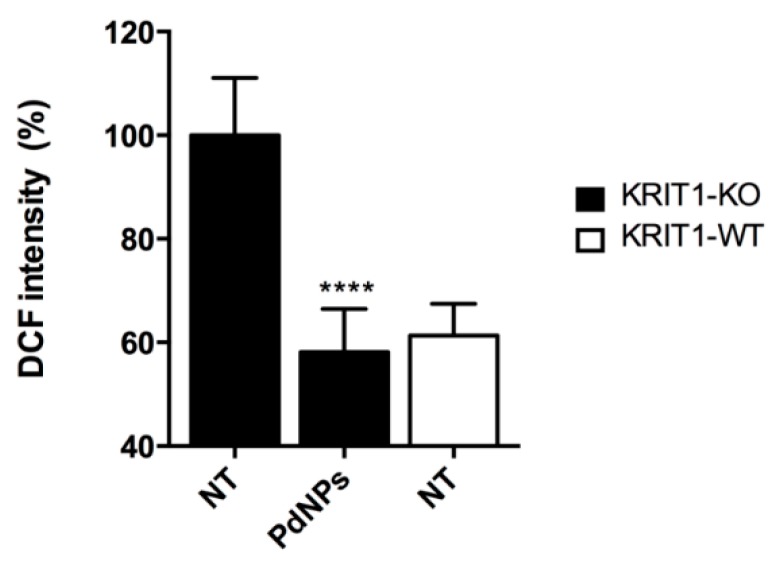
Intracellular levels of Reactive Oxygen Species (ROS) of untreated KRIT1-KO MEF cells (NT), KRIT1-KO cells treated with 25 μg/mL PdNPs for 48 h (PdNPs), and untreated KRIT1-WT MEF cells (NT), evaluated by DCFH-DA assay. The probe fluorescence intensity for PdNP treated KRIT1-KO cells is reported as a percentage relative to untreated KRIT1-KO cells. Results are reported as mean ± SD. The experiments were repeated three times and performed in triplicate.

Oxidative stress is associated with aging and several pathologies, including cardiovascular diseases, cancer, and neurodegenerative disorders [70]. Moreover, the correlation between oxidative stress and vascular dysfunctions in cerebral pathologies has been clearly demonstrated [71,72,73] as in the case of the major cerebrovascular diseases of genetic origin, CCM. There is evidence that loss-of-function mutations in one of three *ccm* genes, namely *ccm1* (*krit1*), impairs cell ability to regulate mitochondrial homeostasis, leading to ROS accumulation and consequent endothelial cell dysfunctions [74]. The high levels of ROS in *krit1* knock-out (KRIT1-KO) cells, derived from a murine model of the human CCM pathology, makes them an optimal cellular tool [75] to test the properties of PdNPs as antioxidant nanozymes. Therefore, we treated KRIT1-KO mouse embryonic fibroblasts (MEFs) for 48 h with PdNPs (25 μg/mL) and we tested the ROS scavenging activity of the nanozyme by DCFH-DA assay. A statistically significant ROS decrease in KRIT1-KO cells was observed, as compared with untreated ones (Figure 8). Interestingly, ROS levels in treated KRIT1-KO cells reached that of the wild type (KRIT1-WT NT), demonstrating the ability of PdNPs to act as efficient antioxidants for the recovery of the physiological redox balance.

## 3. Discussion

We developed a new method based on aqueous environment and reagents known to be biocompatible, green, or easy to remove after synthesis for the production of citrate-capped PdNPs with size tunability and low polydispersity. To the best of our knowledge, this is the first synthetic protocol for PdNP production, performed in aqueous environment without the use of polymers, surfactants, and other strong capping agents. Toxicological studies showed that PdNPs produced with this method do not cause any detectable cytotoxic effect in vitro, thus suggesting their potential for biomedical applications. Cellular internalization, fate, and analysis of NP stability/oxidation was elucidated by TEM and SEI-XPS, demonstrating their stability within the cellular environment, a pivotal aspect for nanozyme exploitation in nanomedicine. Our biocompatible Pd nanozymes possess high antioxidant activity, due to their “naked” surface (fully available for the catalytic reactions) and high surface-to-volume ratio. Such broad nanozyme properties, closely mimicking the size range and activity of the three main antioxidant enzymes, are efficiently exerted at the intracellular level, thanks to their large cellular uptake and stability against oxidation within the cellular environment, as proven by SEI-XPS. This latter point not only confers them high biocompatibility, but also leaves their catalytic efficiency within the cells virtually unaltered, thus allowing long-lasting antioxidant activity. This is a crucial issue for practical applications of nanozymes, since they should maintain their original physico-chemical properties in the cell environment to properly exert their function. Overall, such intracellular stability along with the small dimensions (<10 nm) and monodispersion provide noble metal nanozymes, such as Pt, Pd, and Au, superior performance over other artificial enzymes such as fullerenes, cerium oxide nanoparticles, or graphene materials, where aggregation and low cellular uptake may significantly impact their in-situ catalytic efficiency. Indeed, we observed that our Pd nanozymes have very efficient antioxidant potential, also at the intracellular level, being able of completely recovering the physiological ROS levels in a model of disorder induced by oxidative stress. These findings pave the way to promising perspectives in nanomedicine for the treatment of pathologies associated to ROS overproduction. The versatile nature of PdNPs makes them promising candidates in the treatment of complex pathologies. Their ability to act as biorthogonal catalysts, i.e., to catalyze chemical reactions in vivo, combined with their antioxidant enzyme-like properties, could be a key asset to develop multifunctional artificial enzymes for the catalysis of complex chemical modifications. However, systematic in vivo studies on animal models will be necessary to fully disclose their applicative potential.

## 4. Materials and Methods

### 4.1. Chemicals

All nanoparticle syntheses were carried out in purified MilliQ water. All glass vessels were washed with aqua regia and purified MilliQ water prior to use. Palladium(II)chloride (PdCl_2_), L-ascorbic acid BioXtra product line, sodium borohydride 99.9%, sodium citrate tribasic dihydrate BioUltra product line, citric acid anhydrous, formic acid, potassium bromide, nitric acid, and acetic acid were purchased from Sigma-Aldrich/ Merck KGaA, Darmstadt, Germany.

### 4.2. Synthetic Protocol of Palladium Nanoparticles (PdNPs)

For the synthesis of Pd seeds, a 0.05 M solution of PdCl_2_ was prepared in 0.1M HNO_3_. The acidic environment is necessary as Pd ions at neutral and basic pH tend to form polynuclear hydroxo complexes (PHC) and precipitate in solution, which in turn strongly affects the growth mechanism [34].

Pd seeds were synthesized as follow: 160 μL of Pd solution were added to 360 mL of MilliQ water at 0 °C, followed by a rapid addition of 17.6 mL solution containing 0.03 M sodium citrate and 2 mM citric acid and 1.1 mL of NaBH_4_ (0.02 M), immediately after dissolution in water. The vessel was sealed and quickly transferred to an oil bath at 100 °C to synthesize smaller seeds, due to a fast reduction of the Pd ions. The reaction was kept for 10 min under magnetic stirring. After the end of the synthesis, the vessel was removed and left to cool under stirring for an hour.

For the synthesis of 4 nm citrate-capped PdNPs (Pd4), a 0.05 M solution of PdCl_2_ was prepared in 0.1 M HNO_3_. 320 μL of this solution were added to 180 mL of MilliQ water at room temperature, followed by a rapid addition of 8.8 mL solution containing 0.03 M sodium citrate and 2 mM citric acid and 2.2 mL of NaBH_4_ (0.02 M), immediately after dissolution in water. The vessel was sealed and moved to an oil bath at 100 °C. The reaction was kept at these conditions for 10 min under magnetic stirring. After the end of the synthesis the vessel was left to cool under stirring for another hour. After cooling, the NPs were purified using 10 K Amicon^®^ Ultra Centrifugal Filters, and stored in 2 mM sodium citrate solution at 4 °C.

For the synthesis of 8 nm citrate-capped PdNPs (Pd8), a 0.05 M Pd ions solution was prepared by dissolving PdCl_2_ in 0.1 M CH_3_COOH. A microwave reactor (Flexiwave Microwave Reactor, Milestone) or a sealed glass container (ACE glass pressure reactor with Teflon cap) were used for the syntheses. Briefly, 4 mL of Pd seeds (synthesized as mentioned above), 79.5 μL of Pd (II) solution (0.05 M), and 500 μL of a solution containing 0.34 M sodium citrate, 0.2 M formic acid, and 0.5 mM L-ascorbic acid were added to 30 mL of MilliQ water. The vessel was then sealed, placed within the microwave chamber (or in an oil bath in the case of sealed glass container) and brought to 105 °C in 5 min. The reaction was held stationary for 10 min and then gradually cooled to room temperature. After cooling, the NPs were washed with 2 mM sodium citrate solution using 10K Amicon^®^ Ultra Centrifugal Filters, and stored at 4 °C for future experiments. The multi-vessel setup enables easy scale-up of the synthetic process.

The NP solution was also tested to be endotoxin-free following the instructions of the commercial test Limulus Amebocyte Lysate (LAL) QCL-1000 ^TM^ (Lonza Ltd, Basel, Switzerland).

### 4.3. Determination of PdNP Concentration

PdNP concentration was determined via Inductively Coupled Plasma Atomic Emission spectroscopy (ICP-AES, Agilent 720/730 spectrometer). Briefly, 10–100 μL of the PdNP solution were dissolved in a strong oxidizing agent (aqua regia) overnight and diluted with MilliQ water to a final volume of 10 mL. Measurements were performed at three different wavelengths (*λ* = 177.648, 203.646, and 214.424 nm). Each measurement was repeated in triplicate.

### 4.4. Transmission Electron Microscopy (TEM) Imaging of PdNPs

PdNP size and shape were determined by using a JEOL JEM 1011 microscope, after depositing a methanol dispersion of PdNPs on a carbon coated grid and lefting to dry under vacuum. The size of PdNPs was obtained measuring the diameter of 300 NPs by ImageJ software.

### 4.5. TEM Imaging of Cellular Internalization of PdNPs

HeLa cells (400,000 cell/well in a 6-well tissue culture plate) after 24 h incubation with PdNPs (50 µg/mL) were fixed in 0.1 M sodium cacodylate buffer at pH 7.4 containing 1.5% glutaraldehyde for 1 h. After this procedure, the cells were post fixed in the buffer described above containing 1% osmium tetroxide and stained with an aqueous solution containing 1% uranyl acetate. After dehydratation in graded series of ethanol, cells were embedded in epoxy resin (Epon 812, TAAB) and sectioned with an ultramicrotome (UC6, Leica) equipped with a diamond knife (Diatome). Projection images were obtained by a transmission electron microscope (JEOL JEM 1011, 100 kV acceleration voltage, 2 Mp charge-coupled device (CCD) camera (Gatan Orius SC100, Gatan/AMETEK Inc, Berwyn, PA, USA)).

### 4.6. X-ray Photoelectron Spectroscopy (XPS) Analysis

XPS measurements were performed according to the method previously described [30].

### 4.7. Cell Cultures

MCF-7 (Human Mammary Gland Adenocarcinoma cells, ATCC HTB-22), HeLa (Human Cervix Epithelioid Carcinoma cells, ECACC), Caco-2 (Human Colon Epithelial cells, ATCC HTB-37) and MEF (Mouse Embryonic Fibroblasts, isolated from the knock-out mouse model of the human CCM pathology) cells [74] were expanded at 37 °C in a humidified atmoshere containing 5% CO_2_ in high glucose Dulbecco’s Modified Eagle’s Medium, DMEM (Sigma-Aldrich), containing 10% (*v*/*v*) Fetal Bovine serum (FBS, Sigma-Aldrich), 100 mg/mL streptomycin and 100 U/mL penicillin (Sigma-Aldrich).

### 4.8. WST-8 Assay

The toxicological effect of increasing concentrations of PdNPs (25, 50, and 100 μg/mL) was assessed on MCF-7, HeLa, and Caco-2 cells by WST-8 (Water Soluble Tetrazolium) assay (Sigma-Aldrich). MCF-7 (5000 cells/well), HeLa (5000 cells/well), and Caco-2 (50,000 cells/well) cells were incubated in a 96-well tissue culture plate (Constar, Corning Inc., NY, USA) containing 100 μL of DMEM/well for 24 h at 37 °C in a humidified atmosphere containing 5% CO_2_. DMEM was then replaced with fresh medium containing PdNPs for 24, 48, and 72 h. After washing cells three times with phosphate-buffered saline (PBS) with Ca^2+^ and Mg^2+^, cells were incubated with DMEM containing 10% WST-8 for 1 h. Cell viability was recorded by using an Infinite 200 Pro plate reader. Data were normalized with respect to untreated cells (Ctrl). Results are reported as mean ± SD.

### 4.9. LDH Assay

Membrane damage in HeLa, MCF-7, and Caco-2 cells exposed to increasing doses of PdNPs (25, 50, and 100 μg/mL), was obtained by quantifying the release of lactate dehydrogenase (LDH), a product of lipid peroxidation. HeLa (5000 cells/well), MCF-7 (5000 cells/well), and Caco-2 (50000 cells/well) cells were seeded in a tissue culture-treated 96-well plate (Constar) containing 100 μL of complete DMEM and incubated at 37 °C in a humidified atmosphere containing 5% CO_2_ for 24 h. DMEM was then replaced with fresh medium containing PdNPs and cells were incubated for 24, 48, and 72 h. Afterwards, the LDH assay was performed by using the CytoTox-ONE homogeneous Membrane Integrity Assay reagent (Promega Corporation, Madison, WI, USA). Results were recorded by using an Infinite 200 Pro plate reader. Data were normalized with respect to cells treated with lysis buffer in the same conditions (positive control, P, expressed as 100%). Results are reported as mean ± SD.

### 4.10. DCFH-DA Assay

To evaluate the effect of PdNPs on the ROS homeostasis, HeLa (20,000 cells/well), and Caco-2 (100,000 cells/well) cells were seeded in a tissue culture-treated 96-well plate (Constar) containing 100 μL of complete DMEM and incubated at 37 °C in a humidified atmosphere containing 5% CO_2_ for 24 h. DMEM was then replaced with complete medium containing 25, 50, and 100 μg/mL PdNPs and cells were incubated for 24 h. After washing cells three times with PBS with Ca^2+^ and Mg^2+^, they were incubated at 37 °C with 5 μM 2′,7′-Dichlorofluorescein diacetate (DCFH-DA, Sigma-Aldrich) in the same buffer for 30 min. The probe fluorescence intensity was measured by an Infinite 200 Pro plate reader, using a 480/520 nm filter set. Data were normalized with respect to cells treated with 1 M hydrogen peroxide (H_2_O_2_) for 10 min in the same conditions (P). Results are reported as mean ± SD.

To investigate the intracellular ROS scavenging effects of PdNPs, MEF cells (15,000 cells/well) were seeded in a tissue culture-treated 96-well plate (Constar) containing 100 μL of complete DMEM and incubated at 37 °C in a humidified atmosphere containing 5% CO_2_ for 24 h. DMEM was then replaced with fresh medium containing PdNPs at concentration of 25 μg/mL and cells were incubated for 48 h. After washing cells three times with PBS with Ca^2+^ and Mg^2+^, they were incubated with 5 μM DCFH-DA (Sigma-Aldrich) in the same buffer for 30 min at 37 °C. The probe fluorescence intensity was measured by an Infinite 200 Pro plate reader, using a 480/520 nm filter set. Results were normalized with respect to untreated cells (KRIT1-KO NT). Results are reported as mean ± SD.

### 4.11. Intracellular Uptake of PdNPs

PdNP cellular uptake was measured using ICP-AES (Agilent 720/730 spectrometer) analysis. HeLa (300,000 cells/well) and Caco-2 (1,800,000 cells/well) cells were seeded in a tissue culture-treated 6-well plate (EuroClone SPA, Milan, Italy) containing complete DMEM and incubated at 37 °C in a humidified atmosphere containing 5% CO_2_ for 24 h. DMEM was then replaced with fresh medium containing 50 μg/mL of PdNPs and cells were incubated for 24 h. After washing cells three times with PBS without Ca^2+^ and Mg^2+^, they were harvested using trypsin-EDTA solution. Cell number was measured by a TC10 automatic cell counter (Bio-Rad). After a freeze/thaw lysis process, cell digestion was conducted overnight in CEM Discover SP Microwave synthesizer using fresh aqua regia. The solution was diluted 10 times with MilliQ water, and intracellular amount of Pd was then analyzed by ICP-AES. Each experiment was performed in triplicate.

### 4.12. Antioxidant Activity of PdNPs

Peroxidase (HRP)-like activity of PdNPs was studied by monitoring the oxidation of 3,3′,5,5′-tetramethylbenzidine (TMB, BD Biosciences) at 652 nm for 10 min with a 2 s interval, by the NanoDrop OneC spectrophotometer (Thermo Fisher Scientific Inc., Waltham, MA, USA) equipped with a 1 cm path length cell. The reaction was performed in pH 4.7 acetate buffer, in the presence of 200 mM H_2_O_2_ and 0.5 mM TMB, at room temperature. PdNPs were added as catalyzers at concentration of 0.05 μg/mL. Water was used instead of NPs in the Ctrl sample.

Catalase (CAT)-mimetic activity of PdNPs was evaluated by quantifying the residual H_2_O_2_ (%) after incubation with the nanomaterial, by PeroxiDetect Kit (Sigma-Aldrich). A 40 μM solution of H_2_O_2_ was incubated with a solution of PdNPs containing 0.5 μg/mL of Pd, at room temperature for 0, 30, 90, 180, 300, and 420 min. After the incubation time, the residual H_2_O_2_ was recorded by plate reader. Results were compared with 40 μM H_2_O_2_ measurements over time in absence of catalyzers.

Superoxide ions scavenging ability of PdNPs was evaluated by SOD Assay Kit-WST (Sigma-Aldrich). Superoxide dismutase (SOD) activity was recorded by Infinite 200 Pro plate reader after 20 min of incubation at 37 °C, in presence of 0.05, 0.5, and 5 μg/mL PdNPs.

Antioxidant properties of PdNPs of 8 nm were compared to those of 6-hydroxy-2,5,7,8-tetramethylchroman-2-carboxylic acid (Trolox) by RANDOX assay kit (Randox Laboratories Ltd., Crumlin, UK), following manufacturer’s instructions. PdNPs and Trolox were used at concentration of 77 nM and 2.22 mM, respectively. Water was used as control.

### 4.13. Statistical Analysis

Values are expressed as mean ± SD of three experiments. Statistical analyses were obtained by GraphPad Prism 7 statistical analysis software (GraphPad Prism version 7.04 for Windows, GraphPad Software, San Diego, CA, USA) applying the analysis of variance (ANOVA), using Bonferroni post-test for multiple comparisons. Results were considered statistically significant for *p*-values < 0.05 (* = *p* < 0.05, ** = *p* < 0.01, and *** = *p* < 0.001).

## Supplementary Materials

The following are available online at https://www.mdpi.com/2079-4991/10/1/99/s1, Figure S1: (a) TEM analysis of HeLa cells incubated with Pd8 (a–b) and Pd4 (c–e). (a) is a low magnification projection image of late endosome/phagosome compartments containing Pd8. (b) is a higher magnification of the boxed region in (a). (c–e) are higher magnifications of endosome/phagosome compartments containing Pd4. Scale bars in b-e are 100 nm, Figure S2: XPS survey spectrum of HeLa cells after exposure to PdNPs for 48 hours (a) before and (d) after 300 s of argon sputtering time. The enlarged regions between 225 and 55 eV are presented in (b) and (e). High-resolution spectra of Pd 3d region before and after argon sputtering are presented in (c) and (f) respectively, Figure S3: Relative percentage signal intensities for different Argon sputtering time of Pd (0), Pdox_1_, Pdox_2_, Pdox_pr_ recorded on HeLa cells after exposure to Pd NPs for 48 hours, Figure S4: PdNP antioxidant properties. Time-dependent absorbance signals at 600 nm of 2,2′-azinobis(3-ethylbenzothiazoline-6-sulfonic acid) radical cation (ABTS^+^)after incubation at 37 °C with 77 nM of Pd8 (grey), 2.22 mM of Trolox (purple), and water (black) in the presence of Metmyoglobin and H_2_O_2_, Table S1: Cellular uptake of Pd4 quantified by ICP-AES in HeLa and Caco-2 cells after treatment with 50 μg/mL of Pd4 for 24 h.
