# Supplementary material for: Intracellular Antioxidant Activity of Biocompatible Citrate-Capped Palladium Nanozymes
Source: Nanomaterials (Basel). 2020 Jan 3;10(1):99. doi: 10.3390/nano10010099 (PMC7023661; doi:10.3390/nano10010099)
Supplement: Supplementary file 1 [file nanomaterials-10-00099-s001.pdf]

Supporting Information

Intracellular Antioxidant Activity of Biocompatible Citrate-Capped Palladium Nanozymes

Mauro Moglianetti [1,†,*], Deborah Pedone [1,†], Gayatri Udayan [1,2], Saverio Francesco Retta [3], Doriana Debellis [4], Roberto Marotta [4], Antonio Turco [5], Simona Rella [5], Cosimino Malitesta [5], Giulia Bonacucina [6], Elisa De Luca [1,*] and Pier Paolo Pompa [1,7,*]

[1] Nanobiointeractions & Nanodiagnostics, Center for Biomolecular Nanotechnologies, Istituto Italiano di Tecnologia, via Barsanti, 73010 Arnesano, Lecce, Italy; deborah.pedone@iit.it (D.P.); gayatriudayan@gmail.com (G.U.)

[2] Department of Engineering for Innovation, University of Salento, Via per Monteroni, 73100 Lecce, Italy

[3] Department of Clinical and Biological Sciences, University of Torino, 10043 Orbassano (Torino), Italy; francesco.retta@unito.it

[4] Electron Microscopy Laboratory, Nanochemistry Department, Istituto Italiano di Tecnologia, via Morego 30, 16163 Genova, Italy; doriana.debellis@iit.it (D.D.); roberto.marotta@iit.it (R.M.)

[5] Dipartimento di Scienze e Tecnologie Biologiche e Ambientali (Di.S.Te.B.A.), Università del Salento, via Monteroni, 73100 Lecce, Italy; antonio.turco@unisalento.it (A.T.); simona.rella@unisalento.it (S.R.); cosimino.malitesta@unisalento.it (C.M.)

[6] School of Pharmacy, Via Gentile III da Varano, University of Camerino, 62032 Camerino, Italy; giulia.bonacucina@unicam.it

[7] Nanobiointeractions & Nanodiagnostics, Istituto Italiano di Tecnologia, via Morego 30, 16163 Genova, Italy

* Correspondce: mauro.moglianetti@iit.it (M.M.); elisa.deluca@iit.it (E.D.L.); pierpaolo.pompa@iit.it (P.P.P.)

† Equally contributing authors.

Table S1. Cellular uptake of Pd4 quantified by ICP-AES in HeLa and Caco-2 cells after treatment with 50 μg/mL of Pd4 for 24 hours.

PdNPs	HeLa (pg Pd/cell)	CaCo-2 (pg Pd/cell)
Pd4	0.245 ± 0.023	0.026 ± 0.003

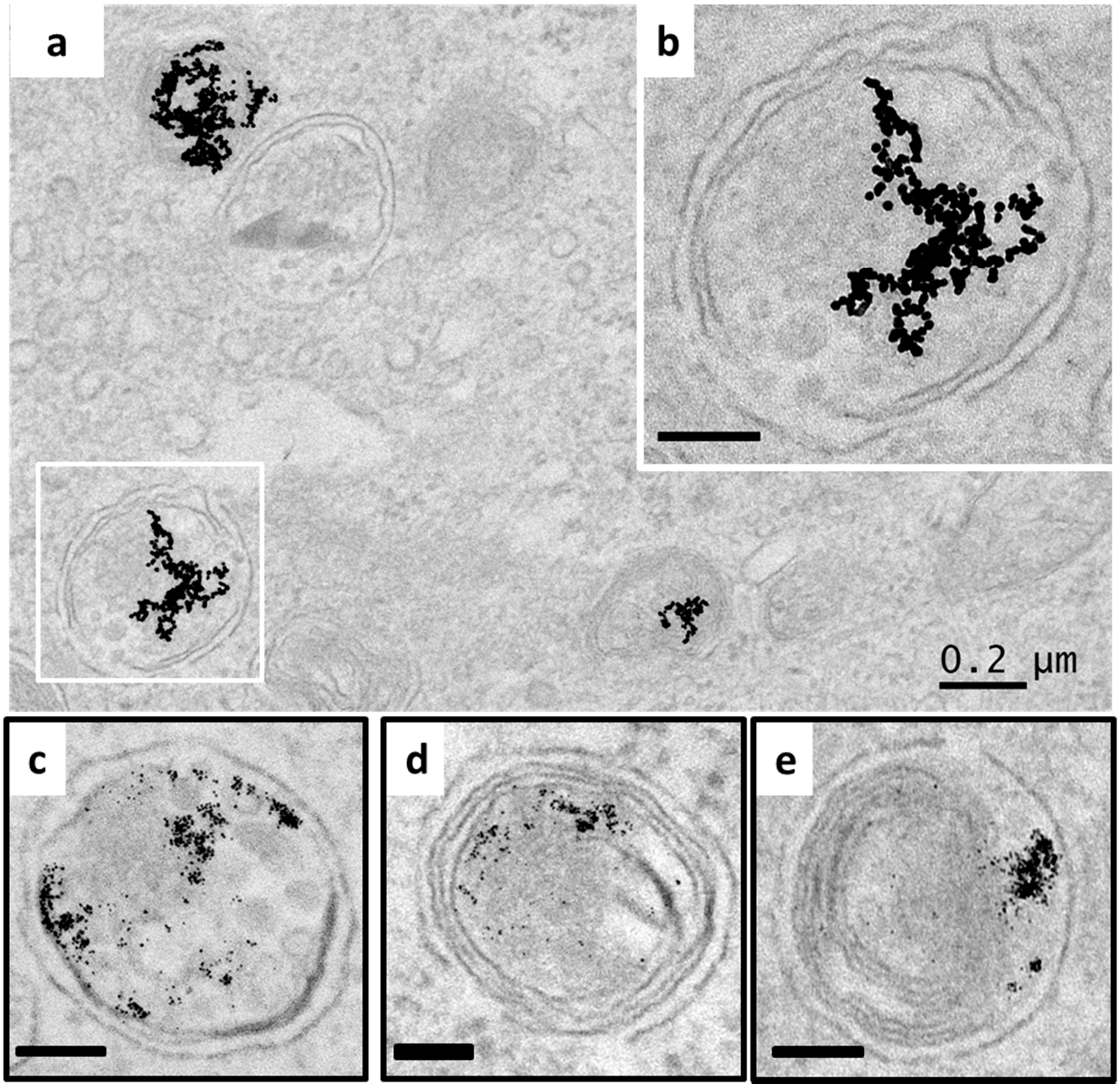

Figure S1. (a) TEM analysis of HeLa cells incubated with Pd8 (**a**–**b**) and Pd4 (**c**–**e**). **a**) is a low magnification projection image of late endosome/phagosome compartments containing Pd8. **b**) is a higher magnification of the boxed region in (**a**). (**c**–**e**) are higher magnifications of endosome/phagosome compartments containing Pd4. Scale bars in b-e are 100 nm.

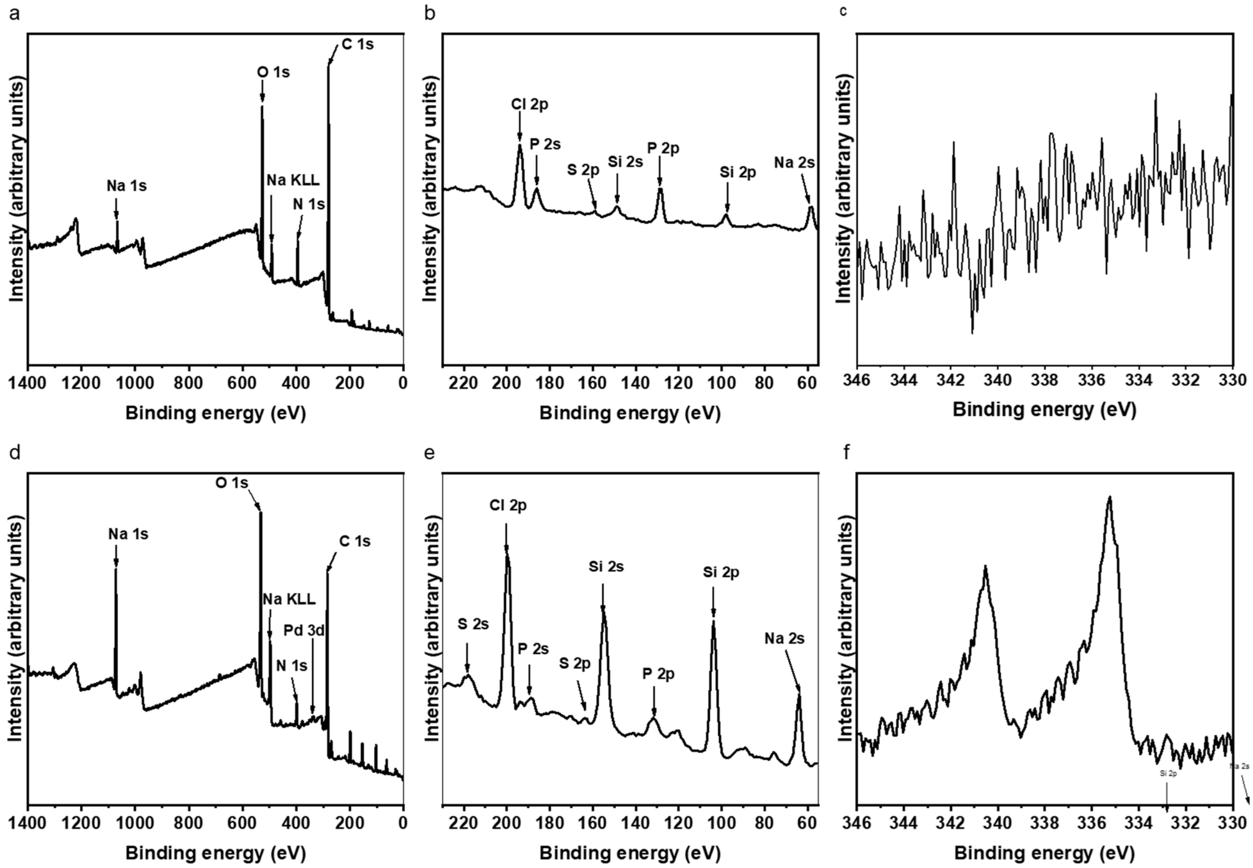

Figure S2. XPS survey spectrum of HeLa cells after exposure to PdNPs for 48 hours (**a**) before and (**d**) after 300 s of argon sputtering time. The enlarged regions between 225 and 55 eV are presented in (**b**) and (**e**). High-resolution spectra of Pd 3d region before and after argon sputtering are presented in (**c**) and (**f**) respectively.

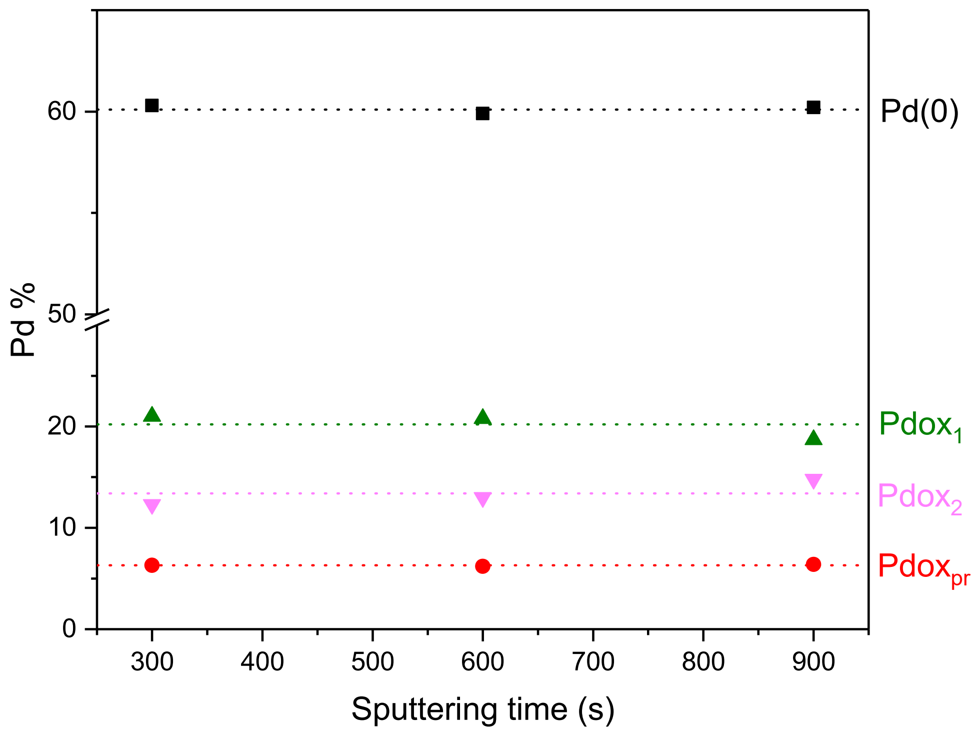

Figure S3. Relative percentage signal intensities for different Argon sputtering time of Pd (0), $Pdox_1$, $Pdox_2$, $Pdox_{pr}$ recorded on HeLa cells after exposure to Pd NPs for 48 hours.

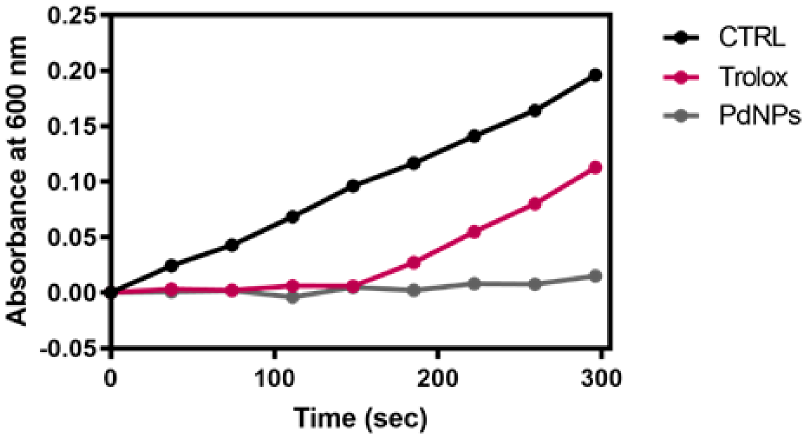

Figure 4. PdNP antioxidant properties. Time-dependent absorbance signals at 600 nm of 2,2′-azinobis-(3-ethylbenzothiazoline-6-sulphonate) (ABTS +) after incubation at 37 °C with 77 nM of Pd8 (grey), 2.22 mM of Trolox (purple), and water (black) in the presence of Metmyoglobin and H_2O_2.
